# A role of Pumilio 1 in mammalian oocyte maturation and maternal phase of embryogenesis

**DOI:** 10.1186/s13578-018-0251-1

**Published:** 2018-10-19

**Authors:** Winifred Mak, Jing Xia, Ee-Chun Cheng, Katie Lowther, Haifan Lin

**Affiliations:** 1 Yale Stem Cell Center, Yale University School of Medicine, 10 Amistad Street, New Haven, CT 06519 USA; 2 Department of Cell Biology, Yale University School of Medicine, New Haven, CT 06519 USA; 3 Division of Reproductive Endocrinology and Infertility, Department of Obstetrics, Gynecology, and Reproductive Sciences, Yale University School of Medicine, New Haven, CT 06519 USA; 4 Department of Genetics, Yale University School of Medicine, New Haven, CT 06519 USA; 5 SIAIS and School of Life Science and Technology, Shanghai Tech University, Shanghai, China; 6 University of Connecticut Health Center, Farmington, CT 06030 USA; 7 Women’s Health Department, Dell Medical School, Medical Park Tower, 1301 W. 38th Street, Suite 705, Austin, TX 78705 USA

**Keywords:** Post-transcriptional regulation, Oocyte maturation, Preimplantation embryo

## Abstract

**Background:**

RNA binding proteins play a pivotal role during the oocyte-to-embryo transition and maternal phase of embryogenesis in invertebrates, but their function in these processes in mammalian systems remain largely understudied.

**Results:**

Here we report that a member of the Pumilio/FBF family of RNA binding proteins in mice, Pumilio 1 (*Pum*1), is a maternal effect gene. The absence of maternal PUM1 in the oocyte does not affect meiotic maturation but leads to abnormal preimplantation development. Furthermore, genome-wide transcriptome analysis of oocytes and embryos revealed that there is a concomitant perturbation of the mRNA milieu. Of note, putative PUM1 mRNA targets were equally perturbed as non-direct targets, which indicates that PUM1 regulates the stability of maternal mRNAs both directly and indirectly. We show *Cdk1 mRNA,* a known PUM1 target essential for meiosis and preimplantation development, is not degraded appropriately during meiosis, leading to an increase in CDK1 protein in mature oocytes, which indicates that PUM1 post-transcriptionally regulates *Cdk1* mRNA; this could partially explain the observed abnormal preimplantation development. Furthermore, our results show that maternal and zygotic PUM1 are required for postnatal survival.

**Conclusions:**

These findings indicate that PUM1 is essential in the process of cytoplasmic maturation and developmental competence of the oocyte. These results reveal an important function of maternal PUM1 as a post-transcriptional regulator during mammalian embryogenesis.

**Electronic supplementary material:**

The online version of this article (10.1186/s13578-018-0251-1) contains supplementary material, which is available to authorized users.

## Background

In many organisms, embryogenesis starts before the activation of the zygotic genome. This maternal phase of embryogenesis lays the foundation for subsequent development and has been well studied in model organisms such as *Drosophila*, *C. elegans*, and *Xenopus*. However, maternal control of embryogenesis in mammals has not been as extensively explored. In mice, the oocyte-to-embryo transition is strictly under maternal control and involves the transformation of a fully grown differentiated oocyte to a totipotent early embryo. Several key events occur during oocyte-to-embryo transition: (1) oocyte maturation that involves both the meiotic maturation of germinal vesicle (GV) to a metaphase II (MII) nucleus (nuclear maturation) and the acquisition of developmental competence (cytoplasmic maturation; [1]); (2) fertilization of MII oocyte by a sperm; (3) zygote formation; (4) first cell division from one to two-cell embryos; (5) maternal–zygotic transition (MZT) at the late two-cell stage during which the zygotic genome is activated to take over the maternal regulation.

In mice, RNA synthesis in the oocyte gradually slows down during the oocyte growth phase so that the full-grown oocyte is transcriptionally silent. This full-grown oocyte will undergo meiotic maturation in which it enters M-phase, completes its meiotic division and is arrested at metaphase II [2]. Meanwhile, degradation of maternal mRNA is initiated during oocyte maturation to erase the oocyte identity [3, 4]. After fertilization, a low level of transcription resumes towards the end of the one-cell stage, called minor zygotic genome activation (ZGA) [5, 6]. Following on from minor ZGA is the major wave of transcription occurring at the two-cell stage, which signifies major ZGA [7]. Thus, there is an extended period of transcriptional silencing during which post-transcriptional mechanisms are vitally essential to enable the successful transformation of the oocyte to an embryo.

RNA binding proteins play a pivotal function during the oocyte-to-embryo transition as they can orchestrate mRNA decay and translational regulation. In invertebrates, several families of RNA binding proteins are known to have reproductive functions: the family of cytoplasmic polyadenylation element binding proteins (CPEB) [8], polyA binding proteins (PABP) [8], and the family of Deleted in Azoospermia (DAZ) [9–11]. For example, *Cpeb1* and *Dazl* in mammals have been shown to play essential roles during germline development [12, 13], oocyte maturation [14], and early preimplantation development [4].

The Pumilio/FBF family (PUF) of proteins are mRNA binding proteins that bind to a consensus sequence called Pumilio response element (PRE) in the 3′UTR of target mRNAs and mediate translational repression and/or mRNA decay [15, 16]. The PUF proteins have highly conserved functions during gametogenesis and embryogenesis in a diverse range of organisms such as *Drosophila, C. elegans, Xenopus* and mouse [17–21]. The PUF family founding member, Pumilio (PUM), was initially discovered as a maternal effect gene regulating anterior–posterior patterning in *Drosophila* [22, 23]. A PUM protein can bind to many mRNAs simultaneously to regulate their expression in *trans* in a coordinated fashion, forming a regulon as proposed by Keene et al. [24, 25]. For example, *Drosophila* PUM binds to more than 600 mRNAs that are enriched in embryogenic functions and are translationally repressed or degraded [26]. In *Drosophila*, PUM represses mRNA translation, accelerates mRNA deadenylation and antagonizes PABP [27]. The N-terminal region of the *Drosophila* PUM possesses the translational repressor function, and its C-terminal region binds to mRNA and presumably mediates mRNA destabilization [28].

In *Xenopus*, there are two PUF proteins, PUM1 and PUM2. PUM1 binds to CPEB and negatively regulates translation of *Cyclin*-*B1* mRNA, such that the injection of the anti-PUM1 antibody into oocytes accelerates production of Cyclin B1 and oocyte maturation [21]. Moreover, *Xenopus* PUM2 is also known to be involved in oocyte maturation by interacting with DAZL and regulating the translation of RINGO/Spy [29]. In *Xenopus* oocytes, PUM1 and PUM2 exist in two separate protein complexes with proteins such as CPEB and DAZL and likely regulate different mRNA pools during oocyte maturation [30]. Decreasing levels of PUM1 and PUM2 accelerate oocyte maturation [30].

In the mouse, polysome-microarray analysis of GV and MII oocytes by Chen and colleagues revealed several binding motifs in the 3′UTR of mRNAs activated during oocyte maturation; these included the consensus for CPE, DAZ, Musashi and PUF families [4]. From this work, it can be hypothesized that mammalian PUM proteins are involved in oocyte maturation. In support of this hypothesis, Chen et al. showed that injecting antisense morpholino oligonucleotide against *Pum2* mRNA resulted in a 50% decrease in meiotic progression [4]. However, this is in contrast to its function in *Xenopus*, demonstrating the uniqueness of PUM function in mammals. This prior work shows that mammalian PUM proteins could have a putative function during nuclear maturation [4]. Both PUM1 and PUM2 are present in mammalian oocytes and granulosa cells [20, 31], which supports a role of PUM during nuclear maturation. However, the published data do not address whether PUM is involved in cytoplasmic maturation that occurs alongside meiotic maturation. Cytoplasmic maturation includes a series of molecular and biochemical events which establishes an oocyte’s developmental competence to be fertilized and to support embryonic development [1]. The role of PUM in these processes is yet to be determined. Furthermore, after fertilization, *Pum1* begins to be transcribed at the two-cell stage and reaches maximal levels at the four-cell stage, whereas *Pum2* is maximally transcribed at two-cell stage [32]. Given the transcription pattern of mammalian *Pum* genes, it is clear that maternal PUM is the sole source of PUM in one-cell embryos.

We previously showed that the murine PUM1 is essential for both oogenesis and spermatogenesis [19, 20], whereas PUM2 does not have a significant function in germline development [20, 31]. We showed that in female mice, PUM1 is vital for the establishment of the primordial follicle pool [20]. *Pum1*^−*/*−^ females are defective in primordial folliculogenesis, leading to a significant reduction in primordial follicle count. Furthermore, we showed that the absence of PUM1 causes a decrease in the number of viable ovulated oocytes and therefore a decline in the number of two-cell embryos [20]. To continue this study, we sought to understand the role of maternal and zygotic PUM1 during the oocyte-to-embryo transition. In this paper, we show maternal PUM1 is dispensable for nuclear maturation but is required for cytoplasmic maturation. In addition, using genome-wide transcriptome analysis of GV, MII and two-cell embryos, we provide a comprehensive view on the role of PUM1 in regulating mRNA stability during these developmental transitions, including that of *Cdk1* mRNA, a known direct PUM1 target that plays a critical role during oocyte maturation and preimplantation development. Furthermore, we show that the absence of both maternal and zygotic PUM1 leads to early postnatal lethality. Our study uncovers the function of mammalian PUM1 during oocyte-to-embryo transition.

## Methods

### Contact for reagent and resource sharing

All reagents and mice are freely available to other investigators by contacting Dr. Haifan Lin (Haifan.lin@yale.edu).

### Experimental animals

All transgenic mice were on a mixed 129/B6 background. *Pum1*^−*/*−^ (global PUM1 knockout) used in this study were previously characterized by Chen et al. [19]. *Pum1*^+*/*+^ mice were littermates of *Pum1*^−*/*−^ mice. Females used in the breeding experiments were between 6 and 8 weeks of age. Male breeders aged 8–10 weeks were fertility tested to have at least one litter before use in experiments. Animals used in these studies were maintained and euthanized according to the principles and procedures described in the NIH Guide for the Care and Use of Laboratory Animals. These studies were approved by the Yale University Institutional Animal Care and Use Committee and conducted in accordance with the specific guidelines and standards of the Society for the Study of Reproduction.

### Oocyte in vitro maturation experiments

To collect GV stage oocytes, ovaries were obtained from 6- to 8-week-old wild-type (WT) and *Pum1*^−*/*−^ (KO) mice 44–48 h after intraperitoneal injection of 5 IU pregnant mare serum gonadotropin (PMSG; Sigma, St. Louis, MO). Ovarian follicles were punctured and GV stage oocytes were collected in MEMα (Life Technologies, Grand Island, NY) supplemented with 20 mm Hepes, 75 μg/ml penicillin G (Sigma), 50 μg/ml streptomycin sulfate (Sigma), 0.1% Polyvinyl Alcohol (PVA; Sigma), and 10 µM milrinone (Sigma) to prevent meiotic resumption. To examine in vitro maturation, oocytes were transferred to MEMα supplemented with 25 mm NaHCO_3_, 75 μg/ml penicillin G, 50 μg/ml streptomycin sulfate, 5% fetal bovine serum (FBS; #12000-022, Life Technologies) and incubated in a humidified atmosphere at 37  °C with 5% CO_2_ and 95% air. Oocytes were assessed after 18 h of culture for germinal vesicle breakdown (GVBD), progression to metaphase II (MII), and expulsion of a polar body (PB).

Progression to MII was assessed by spindle immunofluorescence. Oocytes were fixed in 2% formaldehyde (in buffer containing 100 mm Hepes, 50 mm EGTA, 10 mm MgSO_4_, and 0.2% Triton X-100) and blocked with PBS containing 0.01% Triton X-100, 0.1% PVA, and 3% BSA. Fixed oocytes were incubated in anti-tubulin primary antibody (AbD Serotec, Raleigh, NC) diluted 1:100 in blocking buffer overnight at 4 °C and washed before incubating in secondary antibody (anti-RAT 488, Invitrogen) diluted 1:200 in blocking buffer for 1 h at room temperature. Oocytes were washed with PBS/PVA containing 5 µM SYTOX Orange and imaged on a Zeiss 510 confocal microscope, 40 × 1.2 NA lens using excitation at 488 nm and emission at 530 nm (tubulin) and excitation at 543 nm and emission at 570 nm (SYTOX).

### Oocyte and embryo collection for RNA seq analysis

GV oocytes were obtained for RNA seq analysis as above. In vivo MII oocytes were obtained as follows: 6-week-old females were superovulated with 5 IU PMSG followed 46 h later with 5 IU human chorionic gonadotropin (hCG; Sigma). MII oocytes were collected from the oviducts 15–16 h post-hCG and incubated with hyaluronidase (Sigma; 300 µg/ml) to remove the cumulus cells. The oocytes were then washed in M2 medium (Gibco) and frozen for later use for RNA seq analysis. For two-cell embryo collections, 6-week-old females were superovulated as above and mated with males of proven fertility, at 1.5 dpc, the oviduct was flushed with M2 medium (Gibco), to collect two-cell embryos. A different female was used for each biological replicate for each RNA seq analysis. Each biological replicate contained between 5 and 10 oocytes or two-cell embryos.

### Low-input RNA-seq

Oocytes and two-cell embryos were collected as above and frozen in − 80°. Reverse transcription and cDNA amplification were performed from 5 to 10 oocytes/2 cell embryos using SMARTer Ultra Low RNA kit (Clontech) per manufacturer’s instruction. Sequencing libraries were prepared using Nextera XT DNA Sample Preparation kit (Illumina), according to the manufacturer’s instructions. Libraries were pooled and sequenced on Illumina HiSeq 2000 using single-end 100-base reads.

### Bioinformatics analysis

TopHat (version 2.0.14) was used to align reads to the mouse transcriptome (RefSeq track in UCSC database, version mm10). The parameter values are tuned for processing mammalian RNA-Seq reads (http://tophat.cbcb.umd.edu/). Only the reads that do not fully map to the transcriptome would then be mapped on the mouse genome (version mm10). The reads that did map on the transcriptome would be converted to genomic mappings (spliced as needed) and merged with the genomic mappings in the final tophat output. The command line for Tophat mapping is that tophat2 -o output -p 4 --transcriptome-index mice genes mouse_genome sample1.fastq.gz.

Cufflinks (version 2.2.1) was then used to estimate the abundances of each transcript and tests for differential expression across RNA-Seq samples. It accepts aligned RNA-Seq reads and estimates the relative abundances of the transcripts based on how many reads support each one, taking into account biases in library preparation protocols. Expectation maximization was applied to estimate FPKM (fragment per kilobase of transcript per million reads mapped) scores at both gene and isoform level.

To calculate and plot Spearman correlations for expressed genes across samples, we used functions “corr” and “heatmap.2” in R language. To draw pie charts of genes in different categories, we used Excel functions to show the percentage of a cohort of genes.

To generate the Venn diagrams, the list of genes of interest were inputted in the Venny 2.1 software (available online at https://bioinfogp.cnb.csic.es/tools/venny/). The Venn diagrams were generated by using Venn Plotter (https://omics.pnl.gov/software/venn-diagram-plotter).

### Preimplantation developmental series

Six-week-old females were superovulated with 5 IU PMSG followed 46 h later with 5 IU hCG. The females were then mated with male breeders. Two-cell embryos were collected on 1.5 dpc by flushing the oviducts with M2 media. Blastocysts (3.5 dpc) were collected by flushing the uteri with M2 media.

### Immunofluorescence staining and quantification of fluorescence of MII oocytes

MII oocytes were obtained as described above. Four WT and two KO females were used in two independent experiments. MII Oocytes were fixed in 2% paraformaldehyde and 0.1% polyvinyl alcohol (PVA) in PBS for 1 h at room temperature, permeabilized in 1% Triton X-100 and 0.1% PVA in PBS for 1 h at room temperature, and blocked in 2% BSA and 0.1% PVA in PBS (blocking buffer) for 15 min at room temperature. Then, oocytes were incubated with anti-CDK1 antibody (1:200, ab18, Abcam) in blocking buffer at 4 °C overnight, washed three times for 15 min in PBS with 0.1% Tween 20 at room temperature, and incubated with anti-mouse Alexa Fluor 568 antibody (1:500, ThermoFisher Scientific) in blocking buffer at 4 °C overnight. The oocytes were then washed three times for 15 min in PBS with 0.1% Tween 20 at room temperature, and stained with DAPI (4′,6-diamidino-2-phenylindole). Negative controls were oocytes stained without primary antibody and only secondary antibody. Images were taken using a fluorescent microscope (Zeiss). The images were then analyzed using the Image J software, and the corrected total cellular fluorescence (CTCF) was calculated per oocyte.

### Quantification and statistical analysis

The Chi square test was used in the experiments to compare the percentages of embryos at different developmental stages from the various matings. An unpaired Student t-test was used to compare CTCF of WT and KO obtained after CDK1 immunostaining.

To call differentially expressed genes, we used Cuffdiff package to assess the statistical significance of differential expression at a false discovery rate (FDR) of < 0.05. Briefly, Cuffdiff uses a t-test to calculate the p-value for genes under two conditions. An uncorrected p-value is adjusted to get a false discovery rate (FDR) by Benjamini–Hochberg correction for multiple-testing. A gene is called significantly DE if the FDR value is less than 0.05. DE genes are upregulated or downregulated according to fold change. The other genes with FPKM values greater than 10 are defined as unchanged.

### Sequence data availability

The deep sequencing dataset is deposited in NCBI Sequence Read Archive (SRA) and will be released upon the publication of the current work. The accession link is BioProject 396796 (https://www.ncbi.nlm.nih.gov/bioproject/396796).

## Results

### *Pum1* is a maternal effect gene required for normal preimplantation development

**Fig. 1:**
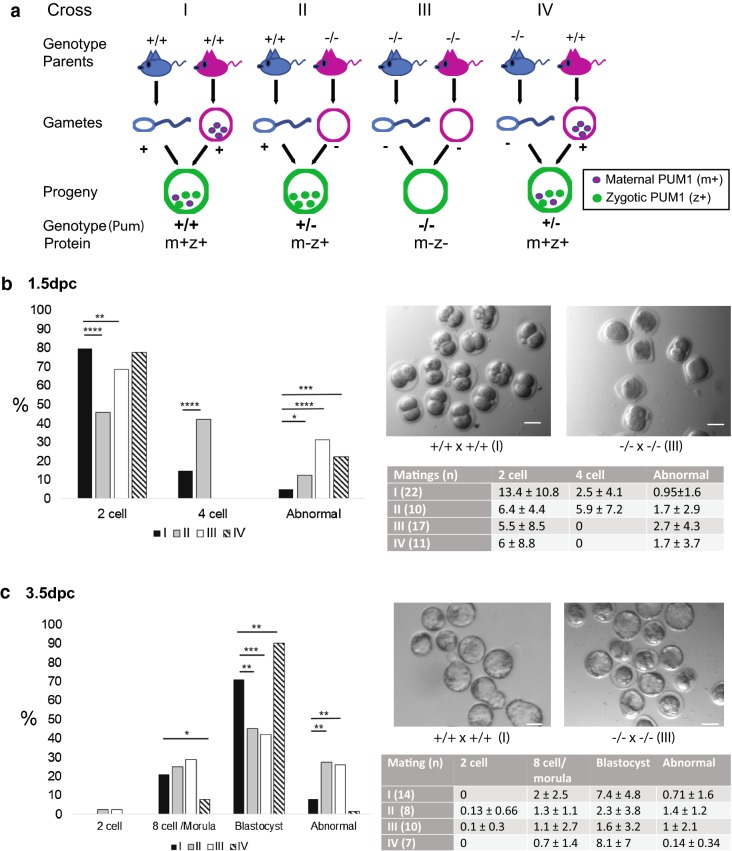
Depletion of maternal *Pum1* leads to abnormal preimplantation development. **a** The schematic of the four crosses used. Of note, zygotic *Pum1* is not expressed maximally until four-cell stage, therefore maternal PUM1 protein is the only source of PUM1 protein during early preimplantation development. Cross I: m+z+ indicates presence of maternal and zygotic PUM1 protein, progeny are *Pum1*^+*/*+^; Cross II: m−z+ indicates maternal PUM1 absent and zygotic PUM1 present, progeny are *Pum1*^+*/*−^; Cross III: m−z− indicates absence of both maternal and zygotic PUM1, progeny are *Pum1*^−*/*−^; Cross IV: m+z+ indicates maternal and zygotic PUM1 present from maternal *Pum1* allele, progeny are *Pum1*^−*/*+^. **b**, **c** Left panels show the percentages of embryos observed at each developmental stage (after excluding unfertilized oocytes). Right upper panels are representative light microscopy images of embryos collected at different time points. Scale bar (white): 500 µm. Right lower panel shows the number of matings for each cross and the mean number (± SD) of each type of embryo seen. Dpc: days postcoitum; 2C and 4C: two-cell and four-cell, embryos, respectively; 8C/M: eight-cell stage embryo and morula; B: blastocyst; abnormal: presumed fragmented embryos; *p < 0.05; **p < 0.01, ***p < 0.001, ****p < 0.0001

To investigate whether maternal PUM1 has a role during preimplantation development, we set up four mating schemes using *Pum1*^+*/*+^/*Pum1*^−*/*−^ male and female mice (Fig. 1a) as follows: *Pum1*^+*/*+^ females with *Pum1*^+*/*+^ males (Cross I); *Pum1*^−*/*−^ females with *Pum1*^+*/*+^ males (Cross II); *Pum1*^−*/*−^ females with *Pum1*^−*/*−^ males (Cross III); *Pum1*^+*/*+^ females with *Pum1*^−*/*−^ males (Cross IV). The females were superovulated, mated with the appropriate males, and embryos were obtained at 1.5 days postcoitum (dpc) and 3.5 dpc. At 1.5 dpc, the number of unfertilized MII oocytes and embryos of different stages was recorded. The fertilization rate (% fertilized MII oocytes/total number of MII oocytes and embryos) observed were as follows: Cross I (+/+ x +/+) 81.3% fertilization; Cross II (−/− x +/+): 76% fertilization; Cross III (−/− x −/−): 43% fertilization and Cross IV (+/+ x −/−): 67.1% fertilization. These results indicate that *Pum1*^−*/*−^ males are associated with a decrease in fertilization rate, consistent with our previous study that *Pum1*^−*/*−^ males have defective spermatogenesis and comprised fertility [19].

To understand whether maternal PUM1 has a function during preimplantation development, we examined the progeny from the four crosses at 1.5 dpc and 3.5 dpc. Figure 1b, c shows the percentage and total numbers of embryos at differing developmental stages at 1.5 dpc and 3.5 dpc. Figure 1b shows that in Cross II and III, where there is a lack of maternal PUM1, there is a perturbation in embryogenesis, with significantly lower percentages of embryos at the two-cell stage in both crosses as compared to the positive control (Cross I). In Cross II, there was a significantly higher percentage of four-cell embryos than Cross I, as well as a greater percentage of fragmented embryos.

Similarly, in Cross III, most of the remaining embryos were fragmented. In Cross IV, where maternal PUM1 is present the proportion of two-cell embryos did not differ from Cross I. Moreover, in 3.5 dpc embryos from Cross II and III, the absence of maternal PUM1 continued to impede their development such that there was a significantly lower percentage of embryos that reached blastocyst stage as well as a higher percentage of fragmented embryos (Fig. 1c).

In contrast, a significantly greater proportion of embryos from Cross IV became blastocysts, and less were fragmented than embryos from Cross I. Taken together, the results show that maternal PUM1 is essential for normal preimplantation development. Moreover, the presence of zygotic PUM1 cannot rescue the delay in preimplantation development due to the lack of maternal PUM1. Therefore, PUM1 is a maternal effect gene.

### Maternal PUM1 is dispensable for oocyte nuclear maturation

**Fig. 2:**
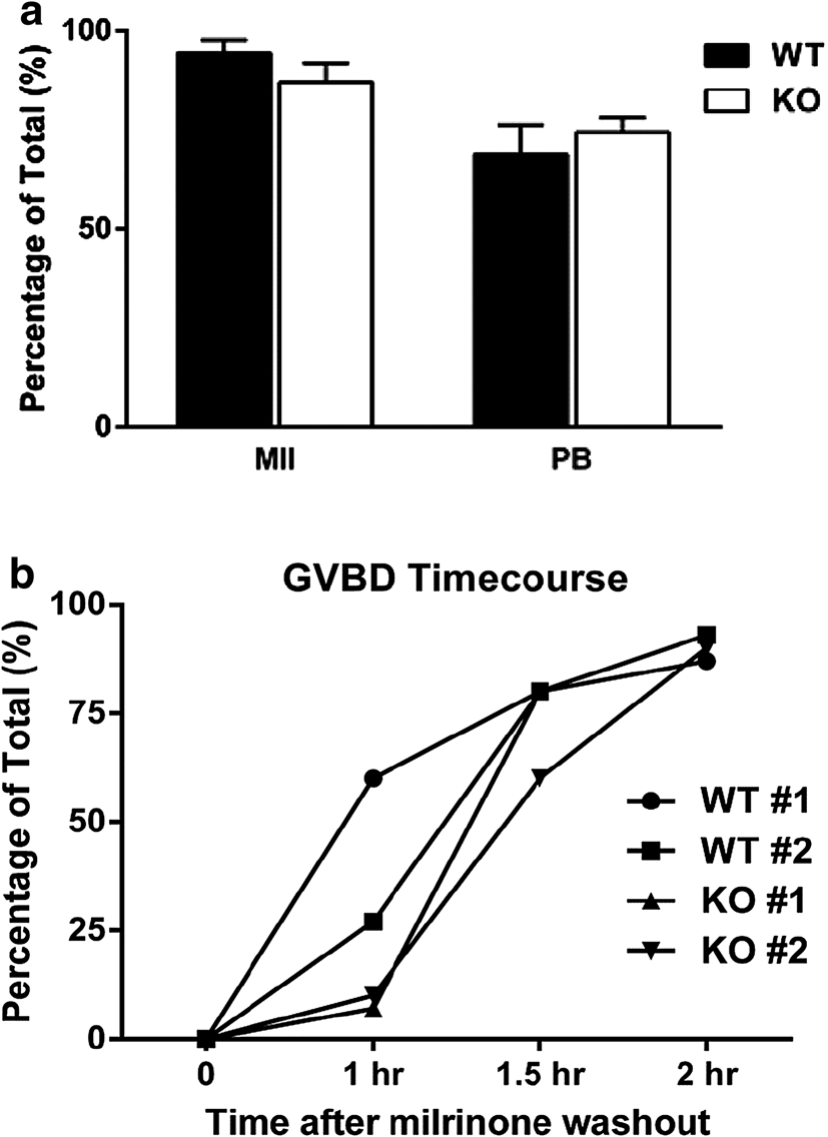
*PUM1* is dispensable for oocyte maturation. **a** Oocyte in vitro maturation using WT (n = 49) and *Pum1*^−*/*−^ (n = 53), three females of each genotype were used. **b** The GVBD time course for *Pum1*^+*/*+^ (WT) (n = 29) and *Pum1*^−*/*−^ (KO) (n = 23) oocytes. Two females of each genotype were used

To investigate whether the maternal effect of PUM1 starts as early as during oocyte maturation, we examined oocyte maturation in *Pum1*^−*/*−^ female mice. Prior studies have shown that a decrease in the mammalian *Pum2* transcript can lead to a delay in meiotic progression of mouse oocytes [4], yet the role of PUM1 in meiotic progression is unknown. Therefore, to understand whether nuclear maturation is normally occurring in *Pum1*^−*/*−^ oocytes, we performed a series of in vitro maturation experiments with *Pum1*^+*/*+^ and *Pum1*^−*/*−^ germinal vesicle (GV) oocytes. 100% of *Pum1*^+*/*+^ and *Pum1*^−*/*−^ GV oocytes underwent germinal vesicle breakdown (GVBD). 94.3% of *Pum1*^+*/*+^ and 86.9% *Pum1*^−*/*−^ GV oocytes matured into MII oocytes, respectively (Fig. 2a). Furthermore, 68.8% of *Pum1*^+*/*+^ and 74.3% *Pum1*^−*/*−^ MII oocytes extruded a polar body and no increase in degeneration of oocytes was observed. Also, GVBD time course was similar between *Pum1*^+*/*+^ and *Pum1*^−*/*−^ oocytes (Fig. 2b). These similar outcomes between *Pum1*^+*/*+^ and *Pum1*^−*/*−^ GV oocytes indicate that, surprisingly, *Pum1*^−*/*−^ GV oocytes have no delay in meiotic maturation. Thus, PUM1 is not required for the process of nuclear maturation during meiotic maturation.

### Maternal PUM1 affects global mRNA dynamics during oocyte maturation

Given that PUM1 depletion does not affect oocyte nuclear maturation yet has a strong maternal effect on preimplantation development, we wondered if maternal PUM1 affected cytoplasmic maturation, a process whereby the oocyte accumulates and stores the necessary RNA/protein to achieve developmental competence. One major pathway used by the oocyte to attain cytoplasmic maturation is selective mRNA degradation [3]. PUM1 could carry out selective mRNA degradation by directly binding to the PRE-consensus on the 3′UTR of target mRNAs. This binding could lead to the shortening of the poly-A tail by PUM1 associated deadenylase complexes and therefore lead to a decrease in mRNA stability. Furthermore, PUM proteins are known to repress translation [33]. However, due to the insufficient material (oocytes/preimplantation embryos), biochemical analysis of translation is not feasible. Therefore, in this study, we focused on the role of PUM1 in regulating target mRNA stability. Our analysis also allowed us to reveal the indirect effects of PUM1 on other mRNAs.

To test the hypothesis that PUM1 affects the mRNA degradation/stabilization during the oocyte-to-embryo transition, we carried out RNA-seq analysis during the period of transcriptional quiescence which occurs from GV oocytes to two-cell embryo stage, since any transcriptome changes seen would reflect the PUM1 regulation of mRNA stability.

**Fig. 3:**
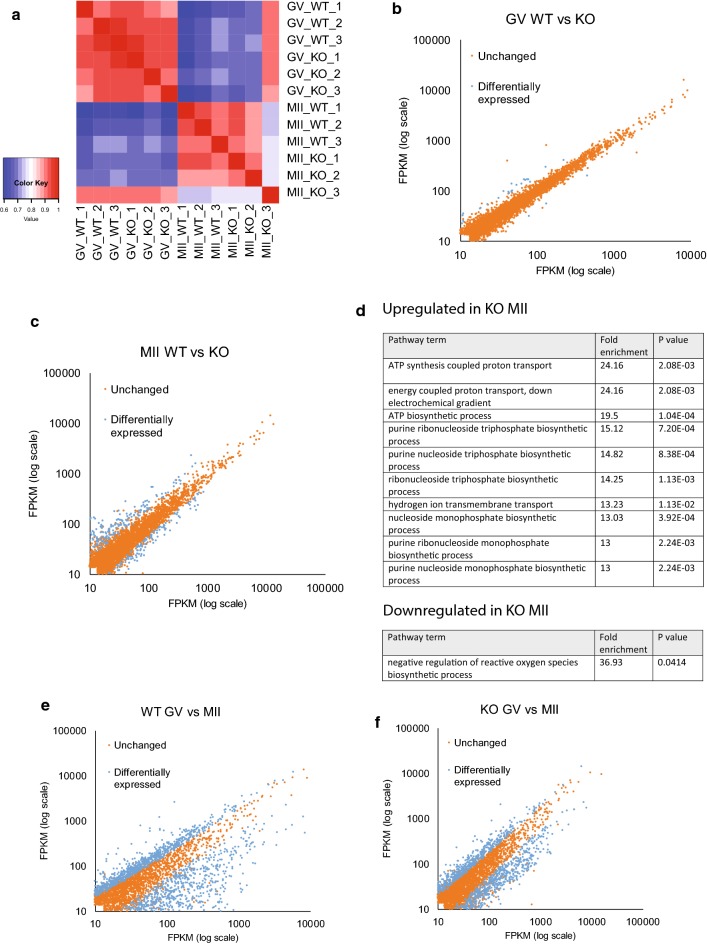
*Pum1*^−*/*−^ MII oocytes show significant changes in transcriptome compared to *Pum1*^+*/*+^ MII oocytes, and lack of PUM1 protein during oocyte maturation leads to a reduction in the dynamics of the mRNA pool. **a** Heatmap of Spearman correlation coefficient of *Pum1*^+*/*+^ (WT) and *Pum1*^−*/*−^ (KO) GV and MII oocytes. **b**, **c** Scatterplots of the unchanged and differentially expressed (DE) genes for WT GV vs. KO GV and WT MII vs. KO MII. **d** Gene ontology analysis using Gene Ontology Consortium website was performed on the genes up/downregulated in KO compared to WT MII oocytes. **e**, **f** Scatterplots of the DE and unchanged genes during *Pum1*^+*/*+^ (WT) and *Pum1*^−*/*−^ (KO) oocyte maturation

To overcome the limited availability of viable oocytes obtainable from *Pum1*^−*/*−^ females, we used a low input RNA-seq approach using oligo-dT primers to study the transcriptome changes during the GV to MII transition in both *Pum1*^+*/*+^(WT) and *Pum1*^−*/*−^ (KO) oocytes. Three biological replicates for each condition were performed. All WT biological replicates showed high correlation (Fig. 3a) and therefore reflected the excellent reproducibility of our biological replicates.

First, we compared the transcriptome of WT and KO GV oocytes. Only 30 differentially expressed genes were found between WT and KO GV oocytes, with seven genes downregulated and 23 genes upregulated (Fig. 3b). This indicates that there were no significant differences in transcriptome between WT and KO oocytes at the GV stage. This result was expected because transcriptional control is the primary mechanism used by growing oocytes to regulate their development. Hence PUM1 is unlikely to affect the transcriptome at this stage.

Next, we investigated whether there were transcriptome differences between WT and KO MII oocytes. 350 genes were significantly differentially expressed by at least twofold in KO MII oocytes when compared to WT MII oocytes, with 248 upregulated and 102 downregulated (Fig. 3c). GO analysis revealed that the genes that are upregulated in KO MII oocytes are enriched for ATP biosynthesis and DNA synthesis. These processes are usually downregulated in MII oocytes as they transit to become metabolically quiescent during metaphase arrest (Fig. 3d). Interestingly, genes that are downregulated in KO MII oocytes are enriched for negative regulation of reactive oxygen species (Fig. 3d). This dysregulation of reactive oxygen species could partly explain an increase in degenerate MII oocytes in *Pum1*^−*/*−^ females found in our prior studies [20]. Together, these data show that, at the MII stage, PUM1 gains a more substantial role in regulating mRNA stability during this period of transcriptional silence.

To estimate how many of the up- and downregulated transcripts are likely direct target of PUM1, we examined their 3′UTR for PRE. Only 23 of the 102 (22.3%) downregulated genes and 31 of the 248 (12.5%) upregulated genes had at least one predicted 3′UTR PRE-consensus sequence. This further indicates that most of the dysregulated mRNAs are due to the indirect effect of PUM1.

We next analyzed the mRNA dynamics during oocyte maturation by examining changes in transcriptome during the GV-to-MII transition in WT and KO oocytes. At the time of this study, preparing a library for RNA-seq using a low number of oocytes was only feasible using oligo dT-priming. Therefore, the interpretation of the following data could be confounded by differences in poly-A tail length. To validate our approach of using low input RNA seq to investigate mRNA dynamics during GV to MII transition, we compared a subset of our data on WT oocytes during the GV-to-MII transition with a microarray study performed by Su et al. [3]. These authors used multi-start primers instead of oligo d-T primers to minimize the effect of poly-A tail length on the analysis and they validated their findings using comprehensive qPCR analysis. They chose five groups of genes to validate with qPCR. We compared the fold change from our analysis with that of the microarray and qPCR validation (Additional file 1: Fig. S1A–D). One of the five groups were granulosa-specific genes *Has2*, *Ptgs2*, *Ptx3*, and *Tnfaip6,* which were either not detectable or had no significant changes across GV to MII transition. As expected, none of these genes were found in our WT dataset. Another group included oocyte-specific genes (Additional file 1: Fig. S1A), in the microarray study, *Bmp15, Gdf9, H1foo, Mater*, and *Zar1.* They showed no significant changes in oocyte maturation, but *Fgf8* was found to be significantly degraded. Interestingly, their qPCR analysis showed significant degradation of all these genes. In our dataset, only *Mater* was found to be significantly degraded, and *Fgf8* was not found in our dataset. Another group of genes were polyadenylated transcripts during oocyte maturation, *Ccndbp1, Gd6pdx, Mos, Plat* and *Spin1* (Additional file 1: Fig. S1B). Su et al. [3] postulated that any method that was biased by poly-A tail length would potentially show a false increase in transcript level in MII such as transcripts which are polyadenylated. In the microarray study, none of the genes showed a significant increase in transcript in MII, and their subsequent qPCR analysis showed all transcripts were degraded. Hence, our data was overall consistent with the microarray, apart from *Mos* which showed a significant increase in transcript level. Su and colleagues also analyzed a group of transcripts that are known to be degraded in MII oocytes, in the microarray all transcripts were significantly lower in MII oocytes and confirmed by their qPCR analysis. Similarly, in our analysis, the same transcripts except *Exosc8* and *Polr2b* were significantly lower in MII. In the microarray there was a group of transcripts which showed ‘upregulation’ in MII oocytes in the microarray study and these were shown to be artifacts of the microarray as their qPCR analysis showed both genes were significantly lower in MII. Taken together, our RNA seq data was consistent with the qPCR data performed by Su et al. (2007) in this group.

In summary, the above analyses verified that our approach is a valid methodology to study the transcriptome of the GV to MII transition and that most of our data are unlikely to be significantly impacted by poly-A tail length despite using oligo-dT primers.

We then analyzed our data for the mRNA dynamics during oocyte maturation. In WT oocyte, 60% (2945) of mRNAs were unchanged, 22% (1070) were significantly lower (> twofold) in MII oocytes than in GV oocytes which represent mRNA degraded, and 18% (868) were significantly higher (> twofold) in MII oocytes which represent transcripts which are stabilized (Fig. 3e). In PUM1-KO oocyte maturation, 73% (3467) of transcripts were unchanged, 15% (710) were degraded, and 12% (544) were stabilized (Fig. 3f). Thus, the absence of PUM1 led to an increase in the number of unchanged transcripts during oocyte maturation and fewer transcripts being degraded or stabilized. These global differences between the transcriptomes of WT and PUM1 mutant oocytes during oocyte maturation indicates that PUM1 has a function in regulating mRNA stability during oocyte maturation.

**Fig. 4:**
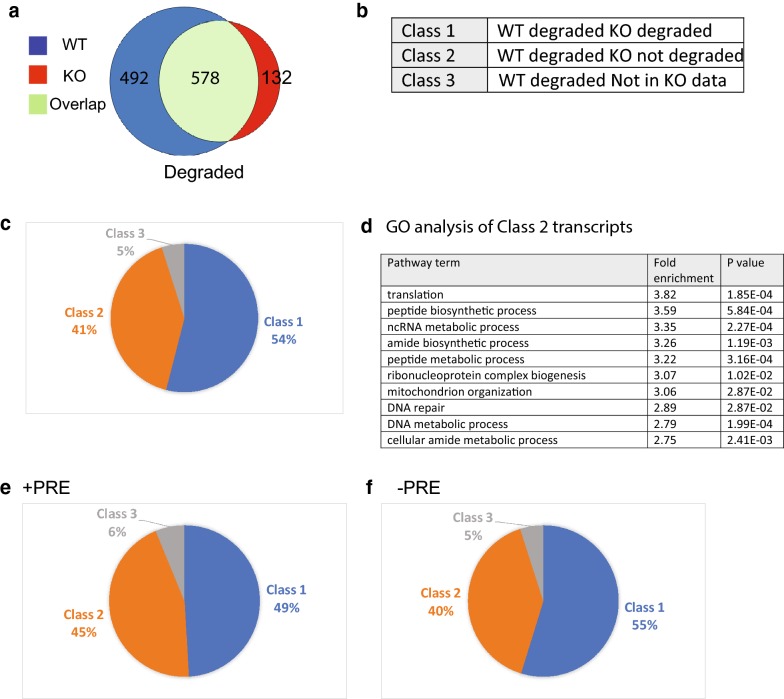
Maternal PUM1 regulates mRNA degradation by direct and indirect mechanisms. **a** The comparison of the mRNA transcripts normally found lower in WT MII (degraded) and the mRNA transcripts found lower in KO MII. **b** The genes were classified according to Class type as described in the table. **c** The percentage of genes found in Class 1–3. **d** GO analysis of the genes in Class 2. **e**, **f** The genes found in **b** were further classified into whether they had a Pumilio-response element (PRE) or without a PRE and the percentages of each class is presented

We then focused our analysis on the transcripts that were degraded during oocyte maturation to understand the biological pathways regulated by PUM1. We compared the transcripts degraded in WT and PUM1-KO oocytes during the GV-to-MII transition (Fig. 4a). We classified the degraded transcripts into three classes (Fig. 4b): Class 1 transcripts underwent normal degradation in KO oocytes during the GV-to-MII transition; Class 2 transcripts did not undergo normal degradation in KO oocytes during the transition; Class 3 transcripts were normally degraded in WT oocytes during maturation but not found in KO oocytes. There were no transcripts found to be normally degraded in WT but activated in KO. Of the 1070 transcripts found to be degraded in WT oocytes during the transition, 578 transcripts (54%) were also degraded in KO oocytes (Class 1). However, 439 transcripts (41%) were no longer degraded in KO oocytes (Class 2), and 53 transcripts (2%) were no longer detected in KO oocytes (Class 3; Fig. 4c). Thus, 41% of genes are not correctly degraded during KO oocyte maturation. This result indicates that PUM1 has a major function in regulating mRNA turnover during oocyte maturation.

GO analysis of the transcripts in Class 2 revealed pathways enriched for translation, protein synthesis, etc. (Fig. 4d). This makes sense because the oocyte should become quiescent during normal oocyte maturation, so processes such as translation and protein synthesis should be winding down. Our results show that lack of maternal PUM1 could dysregulate this developmental programming towards quiescence.

### Maternal PUM1 appears to directly target only a small number of mRNAs during oocyte maturation

To further distinguish whether the dysregulation of mRNA degradation during the maturation of the KO oocytes was a direct and/or indirect consequence of the absence of PUM1, we identified bioinformatically transcripts with putative 3′UTR PRE motifs. Of the 1070 transcripts that are degraded in WT oocytes during maturation, only 161 transcripts (15%) had one or more predicted PRE motif, but 909 (84.9%) of transcripts did not have a PRE. Among the 161 PRE-containing transcripts, 79 of these were Class 1, 72 transcripts were Class 2, ten transcripts were Class 3 (Fig. 4e). Among the 909 transcripts without PRE, 498 transcripts were Class 1, 366 transcripts were Class 2, and 45 transcripts were Class 3 (Fig. 4f). These results indicate that PUM1 may directly degrade a small number of mRNAs, which then lead to an indirect effect on the abundance of many other mRNAs as detected in our analysis.

**Fig. 5:**
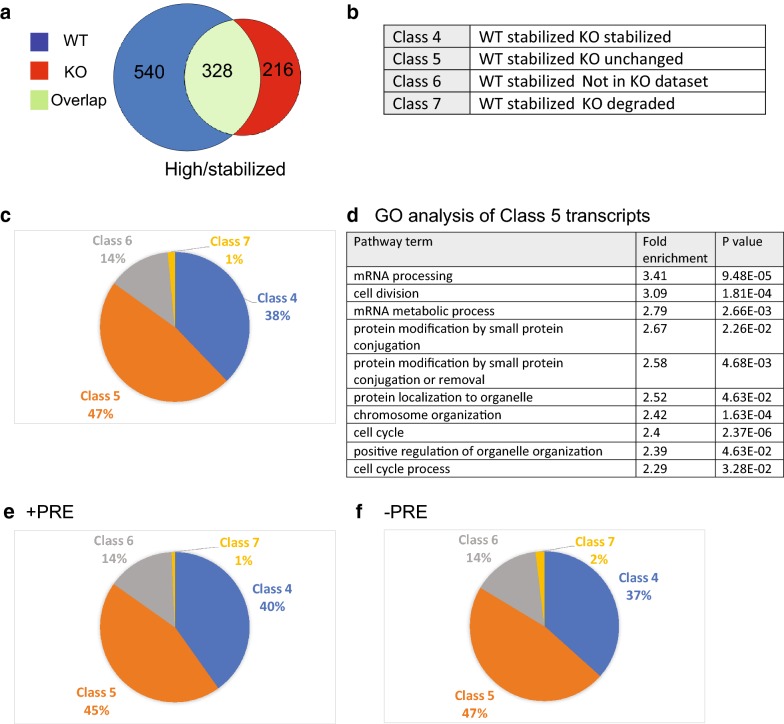
Absence of maternal PUM1 affects transcripts typically stabilized in *Pum1*^+*/*+^ oocyte maturation to a greater extent than those that are typically degraded. **a** The comparison of the mRNA transcripts usually found higher in WT MII (stabilized) and the mRNA transcripts found higher in KO MII. **b** The genes were classified according to Class type as described in the table. **c** The percentage of genes found in Class 4–7. **d** GO analysis of the genes in Class 5. **e**, **f** The genes found in **b** were further classified into those with PRE and without PRE

We then examined the role of PUM1 in transcripts found to be at higher levels in both WT and KO oocytes. Less overlap between WT and KO oocytes were seen in this cohort of transcripts (Fig. 5a). We similarly classified these transcripts into Classes 4–7 (Fig. 5b). Class 4 transcripts are equally stabilized in MII vs. GV in both WT and KO; Class 5 transcripts are dysregulated in the KO oocyte maturation; Class 6 transcripts are stabilized in WT but not in present in the KO dataset; Class 7 transcripts are stabilized in WT but degraded in KO. In Class 4 there were 328 transcripts, 409 transcripts in Class 5, 118 transcripts in Class 6 and 13 transcripts in Class 7 (Fig. 5c). Of note, there was a surprisingly greater number of genes dysregulated in the KO than that of the degraded genes. This suggests a more prominent role of PUM1 in the stabilization of transcript rather than degradation. GO analysis of Class 5 transcripts showed that there was an enrichment for genes involved in mRNA processing, cell division, mRNA metabolic processes (Fig. 5d). This result indicates that the oocyte is stockpiling selective RNAs which will be required during early embryogenesis and that PUM1 has a significant role in regulating this process.

We performed a similar bioinformatic analysis looking at the stabilized transcripts with regards to the presence or absence of PRE. Interestingly, there was a higher number of stabilized transcripts with PRE (289 transcripts 33%) than that in degraded transcripts (15%). 579 (77%) stabilized transcripts had no PRE. Of the 289 transcripts with at least one PRE, 116 transcripts were Class 4, 129 were Class 5, 42 were Class 6 and 2 were Class 7 (Fig. 5e). Of the 579 transcripts with no PRE, there were 212 transcripts in Class 4, 272 in Class 5, 84 in Class 6 and 11 in Class 7 (Fig. 4f). Again, there was no evident enrichment for transcripts with or without PRE being more perturbed by the absence of PUM1.

In summary, our data indicate that maternal PUM1 does have an impact on the global transcriptome during oocyte maturation. PUM1 regulates genes involved in processes that are vital for developmental competence but not meiotic genes or those involved in nuclear maturation. It is still possible, however, that PUM1 regulates the translation of many mRNAs during oocyte maturation by translational regulation, which will not be detected by our analysis.

### Maternal PUM1 affects global mRNA dynamics during early preimplantation development

If maternal PUM1 regulates the stability of maternal mRNAs, we would predict that the pool of mRNAs present from fertilization to maternal–zygotic activation would be affected by the absence of PUM1. Therefore, we performed RNA seq analysis of *m*+*z*+ two-cell embryos from Cross I (*Pum1*^+*/*+^ self-mating), *m*−*z*+ two-cell embryos from Cross II (*Pum1*^−*/*−^ females mating with *Pum1*^+*/*+^ males) and m−z− two-cell embryos from Cross III (*Pum1*^−*/*−^ self-matings) (Fig. 1a). All samples were sequenced in triplicate, which showed good reproducibility of the WT two-cell replicates (Additional file 2: Fig. S2A). The transcriptomes of WT MII oocytes were very different from those of WT two-cell embryos. These data reveal a significant change in transcriptome between MII oocytes and two-cell embryos.

We then compared the transcriptome difference among the three types of two-cell embryos to investigate the function of maternal PUM1 in regulating the transcript dynamics in two-cell embryos. Zygotic PUM1 is not maximally expressed until four-cell stage. Therefore any transcriptome differences prior to this are likely to be mostly under maternal PUM1 control. There were 331 downregulated transcripts and 316 upregulated transcripts when comparing *m*+*z*+ (WT) and *m*−*z*+ (HET) two-cell embryos (Additional file 3: Fig. S3B). There were 199 downregulated/transcripts and 237 upregulated/transcripts in *m*−*z*− (KO) compared to *m*+*z*+ (WT) two-cell embryos (Additional file 2: Fig. S2C). Interestingly, there were less differentially expressed genes when comparing *m*−*z*+ (HET) two-cell and *m*−*z*− (KO) two-cell transcriptomes, with only 120 transcripts downregulated and 115 upregulated transcripts (Additional file 3: Fig. S3D). Because *m*−*z*+ (HET) two-cell and *m*−*z*− (KO) two-cell embryos were more similar in their transcriptome than with *m*+*z*+ (WT) two-cell embryos. These results indicated that maternal PUM1 has a significant influence on the RNA stability even after fertilization.

**Fig. 6:**
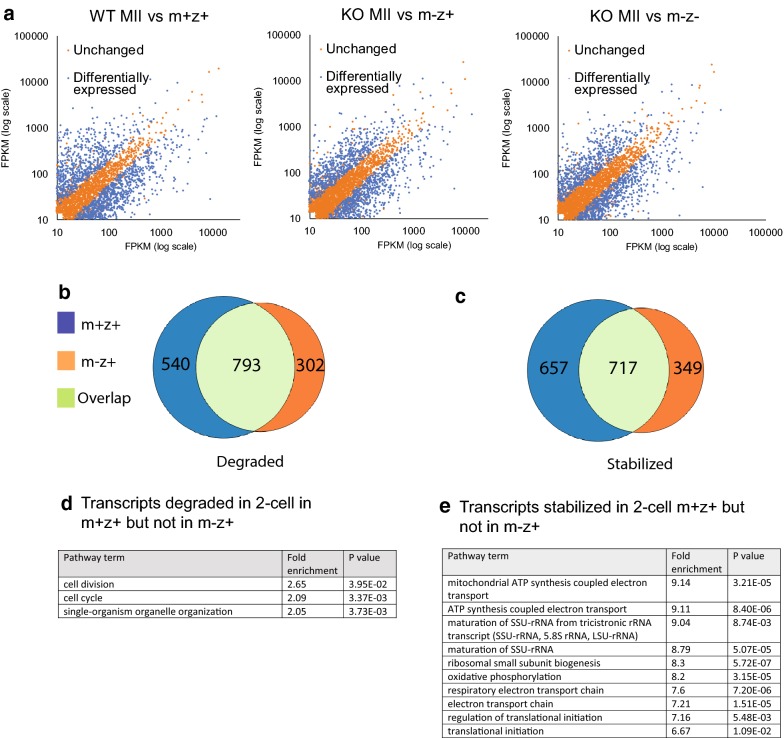
The maternal effect of PUM1 on mRNA milieu continues into early preimplantation development. **a** The scatterplots for comparison of the transcriptome changes during MII to two-cell transition between the different matings. **b** The comparison of the transcripts lower (degraded) in WT MII to m+z+ two-cell embryos transition and KO MII to m−z+ two-cell embryo transition. **c** The comparison of the transcripts higher (stabilized) in WT MII to m+z+ two-cell embryos transition and KO MII to m−z+ two-cell embryo transition. **d**, **e** GO analysis of the transcripts dysregulated in KO MII to m−z+ two-cell embryo transition

To explore the mRNA dynamics during MII to two-cell transition which would be likely to be regulated by maternal PUM1, we compared the transcriptome of WT MII oocytes to *m*+*z*+ two-cell embryos, KO MII to *m*−*z*+ two-cell embryos, and KO MII to *m*−*z*− two-cell embryos. Similar to the oocyte data, lacking maternal PUM1 lead to fewer transcripts being stabilized and degraded (Fig. 6a) and more transcripts remaining unchanged across the developmental transition. Therefore, PUM1’s function in regulating the mRNA pool is consistent from GV to two-cell transition.

To isolate the transcripts that are controlled by maternal PUM1, identification of genes inappropriately regulated during transition from KO MII oocytes to *m*−*z*+ two-cell embryos was performed. 48.8% (793) of transcripts normally degraded during the WT MII-to-*m*+*z*+ transition were also found to be similarly degraded in the KO MII-to *m*−*z*+ transition (Fig. 6b). However, 40.5% (540) of transcripts were not appropriately degraded during the MII-to-2-cell transition in *m*−*z*+ (Fig. 6b). GO analysis of the transcripts that were not appropriately degraded in m−z+ two-cells showed enrichment in genes involved in cell division and cell cycle (Fig. 6d). This result indicates that PUM1 is fine-tuning genes involved in development and interestingly more of m−z+ embryos had accelerated development to four-cell embryos (Fig. 1b).

We then examined how many mRNAs that are normally stabilized during the MII-two-cell embryo transition and how many of such transcripts are affected when maternal PUM1 is depleted. 41.6% (717) of transcripts normally stabilized during the WT MII-to-*m*+*z*+ two-cell transition were similarly regulated in the KO MII-to-*m*−*z*+ two-cell transition (Fig. 6c). However, 38.1% (657) of transcripts that were stabilized during transition from WT MII oocytes to *m*+*z*+ two-cell embryos are no longer stabilized during the KO MII oocytes to *m*−*z*+ two-cell transition (Fig. 6c). GO analysis of these misregulated transcripts revealed enrichment for genes with a function in ATP synthesis and regulation of translation initiation (Fig. 6e). This analysis indicates that maternal PUM1 regulates the stability of transcripts that are important in the conversion of a metabolically quiescent oocyte to a metabolically active two-cell embryo.

In summary, our results show that PUM1 positively and negatively regulates the stability of different maternal mRNAs during early embryogenesis but to a less extent than during oocyte maturation.

### Maternal PUM1 regulates *Cdk1* during the oocyte-embryo transition

**Fig. 7:**
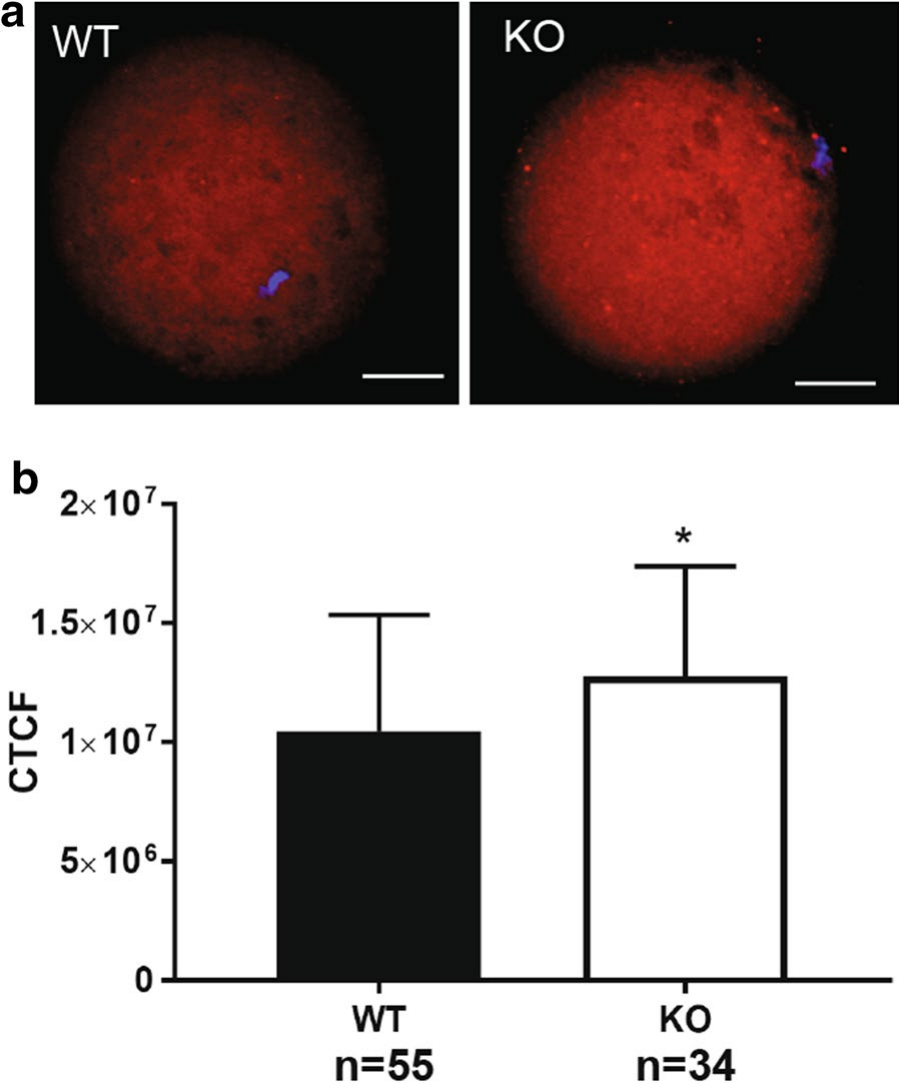
Lack of maternal PUM1 in oocytes causes an increase in CDK1 protein levels. **a** Representative images of the immunostaining of WT (*Pum1*^+*/*+^) and KO (*Pum1*^−*/*−^) MII oocytes with the anti-CDK1 antibody. Scale bar (white): 50 μm. **b** The quantification of the fluorescence signal. n = total number of oocytes scored. CTCF, corrected total cellular fluorescence (units) per oocyte (mean ± SD); WT, *Pum1*^+*/*+^ MII oocytes; KO, *Pum1*^−*/*−^ MII oocytes. * p < 0.05

Next, we focused our analysis on genes that could be direct targets of PUM1 and could contribute to the observed phenotype. Additional file 4: Table S1 shows the list of PRE-containing mRNAs that are differentially expressed between WT and KO MII oocytes. Cyclin-dependent kinase 1 (*Cdk1*) was the most overexpressed candidate gene in KO MII. *Cdk1* mRNA contains one PRE-element in the 3′UTR and was present at much higher levels in KO MII oocytes. *Cdk1* is a member of the Ser/Threonine kinase family and has been shown to be an essential regulator of meiotic resumption in mouse GV oocytes [34] and also required during preimplantation development [35]. During the WT oocyte maturation, the *Cdk1* transcript levels were significantly lower in MII oocytes than in GV (log2(− 6.3) fold change), therefore showing that *Cdk1* transcripts are normally degraded during oocyte maturation. During KO oocyte maturation, the *Cdk1* transcript was decreased but only by log2(− 1.59) in MII. These data indicate that the absence of PUM1 leads to reduced degradation of *Cdk1* transcript. To examine the increase of CDK1 at the protein level, we performed immunostaining of *Pum1*^+*/*+^ and *Pum1*^−*/*−^ MII oocytes with anti-CDK1 antibody (Fig. 7a, b), which showed that *Pum1*^−*/*−^ MII oocytes have a significant increase (20%) in CDK1 protein levels compared to control oocytes.

Interestingly, *Cdk1* is a major ZGA gene, and *Cdk1* null mutant embryos fail to develop into morula and blastocyst stages [35]. Given that these crucial events are tightly regulated, we hypothesized that abnormal *Cdk1* mRNA dynamics during the oocyte-embryo transition could be detrimental to preimplantation development. Therefore, we investigated the dynamics of *Cdk1* from MII to two-cell stage. There was a significantly higher level of *Cdk1* transcript in m+z+ 2 cell embryos as compared to WT MII (log2(7.82)) oocytes. This is expected given it is a major ZGA transcript, whereas comparing KO MII to m−z+ and m−z− two-cell embryos, there was a smaller increase (log2 (3.01) and log2 (3.02) respectively). The levels of *Cdk1* transcript was not different between m+z+, m−z+ and m−z− two-cell embryos. Therefore, the less prominent increase in *Cdk1* transcript in the embryos lacking maternal PUM1 is likely due to suboptimal degradation of *Cdk1* transcript in KO MII oocytes. This effect is then sustained through early preimplantation development. Hence, we speculate that the different *Cdk1* mRNA dynamics during the oocyte–embryo transition in the absence of maternal PUM1 could possibly explain the observed abnormal preimplantation development.

### Both maternal and zygotic PUM1 are essential for postnatal survival

Having shown that maternal PUM1 functions during early preimplantation development, we further investigated whether PUM1 is required after this developmental stage. We collected data on litter sizes and survival in Cross I, II, and III as shown in Additional file 3: Fig. S3. Interestingly, the absence of maternal PUM1 partially affects the postnatal survival of progeny (Cross II). However, the deficiency of both maternal and zygotic PUM1 lead to 100% postnatal lethality of progeny (Cross III). This suggests that both maternal and zygotic PUM1 are both required for postnatal survival.

## Discussion

In this study, we have shown that mammalian *Pum1* is a maternal effect gene required for the successful oocyte–embryo transition. Furthermore, the absence of both maternal and zygotic PUM1 leads to complete perinatal lethality. We showed that deficiency in maternal PUM1 led to dysregulation of the cytoplasmic RNA stores in maturing oocytes and two-cell embryos. Our results reveal that at least part of the developmental function of the maternal PUM1 is achieved via regulating the stability of these mRNAs, in addition to its better-known role in translational regulation. These observations echo well with the role of Pumilio genes in lower organisms as a maternal effect gene in early embryogenesis. Also, we show that PUM1 regulates *Cdk1* transcript post-transcriptionally in oocytes, this could be one putative pathway that underlies the role of PUM1 in the acquisition of oocyte developmental competence leading to normal preimplantation development.

### PUM1 is a maternal effect gene crucial for cytoplasmic maturation and embryogenesis but not required for meiotic maturation

Pum was initially found as a maternal effect gene in *Drosophila* causing anterior–posterior patterning defects [22, 23]. Our study shows that *Pum1* belongs to a select group of mammalian maternal effect genes. A recent review cited 29 mammalian maternal effect genes [36], and *Pum1* shares many similarities to previously reported maternal effect genes such as *Stella* [37], *Atg5* [38] and *Zfp57* [39]. For example, with the absence of maternal PUM1, there is no effect on meiotic maturation, but there is aberrant preimplantation development (similar phenotype to *Atg5/Stella*). Further supporting evidence for PUM1 as a maternal effect gene is that, similarly to m−z− embryos, many of m−z+ embryos still did not reach the blastocyst stage, despite zygotic activation at the two-cell stage. These observations show that the preimplantation lethality phenotype is entirely maternally driven.

Furthermore, the finding that there were more m−z+ embryos proceeding to the four-cell stage than m+z+ embryos indicates the possibility that maternal PUM1 could act to repress major zygotic activation. Therefore, lack of maternal PUM1 allowed more embryos to prematurely divide to the four-cell stage. Finally, when *Pum1*^−*/*−^ oocytes are fertilized with *Pum1*^−*/*−^ sperm, resultant *Pum1*^−*/*−^ pups (lacking both maternal and zygotic PUM1) are born but die shortly after birth, which is reminiscent of the perinatally lethal phenotype of a maternal–zygotic gene, *Zfp57* [39]. This indicates that the absence of zygotic PUM1 has an additive effect during post implantation development.

Surprisingly, the absence of PUM1 in oocytes did not affect nuclear maturation, even though much previous data in lower organisms and limited data in mice suggested that nuclear maturation would be perturbed. One explanation is that PUM2 is still present in the oocytes to compensate for the function of PUM1. Additionally, there could have been selection bias to choose the healthier looking GV oocytes for the in vitro maturation experiments. Even considering this, one can comfortably conclude that PUM1 is not essential for nuclear maturation in GV.

The phenotype seen with the absence of maternal PUM1 is different from other maternal effect genes in which complete two-cell block is usually observed. Of note, PUM2 is still present in the *Pum1* mutant. Therefore, there may also be partial compensation of the maternal PUM1 function by PUM2 as PUM1/2 have overlapping mRNA targets.

### PUM1 acts as a post-transcriptional regulator during oocyte-to-embryo transition

Based on several prior studies of PUM proteins in lower organisms and mammals, PUM1 could regulate both the stability and translation of target mRNAs. For example, *Drosophila* Pum can increase mRNA deadenylation to bring about mRNA destabilization; meanwhile, it also acts as a translational repressor [27, 28]. Prior studies of mammalian PUM in testes [19] and brain [40] have shown that translational regulation is the predominant mechanism in these tissues. Our study shows that maternal PUM1 regulates the stability of a large number of mRNA transcript levels during the oocyte-to-embryo transition, in addition to the translational regulon that may target an overlapping set of mRNAs [24].

Our RNA-seq approach allowed us to understand from a genome-wide perspective whether maternal PUM1 can change transcriptome stability in addition to its known translational regulatory function. The strengths of our RNA seq analysis are that it spans GV to the two-cell stage and we have shown a global perspective of a post-transcriptional mechanism for PUM1 action during these developmental stages. We have attempted to address bioinformatically whether the role of PUM1 towards individual mRNAs is direct via PRE binding or indirect. Ideally, RIP or iCLIP analysis would be the gold-standard to identify direct targets. However, these approaches are not feasible, given such a limited availability of the material. Specifically, our study is the first to revisit RNA degradation during GV to MII transition using RNA seq analysis since the microarray analysis by Su et al. [3].

Our data show that PUM1 is changing the RNA pool in a selective manner. During oocyte maturation, PUM1 silences genes associated with ATP metabolism, protein synthesis during oocyte maturation to enable the oocyte to become metabolically quiescent. Also, PUM1 stabilizes transcripts required for preimplantation development such as genes involved in mRNA processing and cell division. This maternal program needs to be set before fertilization. Once fertilization occurs, in the two-cell embryo, PUM1 again regulates transcripts involved in ATP metabolism, but interestingly, stabilization of these transcripts occurs, in contrast to their change during oocyte maturation. Thus, it is likely that PUM1 can act both as a repressor and activator of same transcripts depending on developmental context. Alternatively, PUM1 is only required to appropriately degrade maternal transcripts before fertilization to enable zygotic activation to take place. As proposed by prior researchers, selective degradation/stabilization of transcripts during oocyte maturation is a vital process required for developmental competence and any disturbances in this process will lead to perturbation of embryonic development [3]. The absence of PUM1 disturbs the mRNA dynamics during these critical developmental stages leading to abnormal preimplantation development.

Our results show that the direct targeting of a large number of mRNAs by the maternal PUM1 leads to the indirect regulation of an even larger population of mRNAs. This occurs during the oocyte maturation phase and progressing into the two-cell stage. Although we did not find any preponderance of transcripts with PRE that were more likely to be dysregulated, there was a tendency for transcripts usually stabilized during the GV to MII transition to be more likely direct targets than during MII to two-cell embryo stage. This result might suggest that the role of PUM1 is predominantly in regulating cytoplasmic maturation in the oocyte and the changes in MII to two-cell are due to indirect effects of the transcripts already dysregulated during oocyte maturation.

### PUM1 regulates *Cdk1* during oocyte-to-embryo transition

Our RNA seq analysis revealed that *Cdk1* is degraded during normal oocyte maturation as it is not required once the MII is arrested. Subsequently, the *Cdk1* transcript level is increased in two-cell embryos partly due to stabilization of maternal *Cdk1* transcripts and/or zygotic genome activation. One explanation for the preimplantation phenotype seen in our study is that *Cdk1* is not appropriately repressed or degraded during oocyte maturation in the absence of maternal PUM1. Therefore, the developmental program is disrupted, leading to the observed abnormalities during preimplantation development. This is consistent with the theory that embryogenesis is tightly regulated and follow a strict temporal sequence of events, i.e. which transcripts are translated and when they are translated. Future studies will elucidate how PUM1 regulates *Cdk1* transcript, i.e., directly through poly-A tail lengthening and shortening or indirectly, for example, *Cdk1* also has a binding site for DAZL so PUM1 may interact with DAZL to indirectly regulate *Cdk1* levels.

### Maternal and zygotic PUM1 are required for postnatal survival

Prior studies showed that the absence of zygotic PUM1 alone could lead to preimplantation lethality. However, this study used a gene-trap strategy which could have off-target effects leading to lethality, and also this group used mutants on a pure B6 background [31], which could have generated synthetic lethality with the *Pum1* mutation. In our study, *Pum1*^−*/*−^ mice in a mixed background of B6/129 are viable, indicating that zygotic PUM1 alone is not essential for embryogenesis. Interestingly, in our study when both maternal and zygotic PUM1 was absent, all the pups died shortly after birth. This result suggests that there are vital genes regulated by both maternal and zygotic PUM1 which are essential for postnatal survival or that the few maternal/zygotic null survivors observed are the exceptions to the rule and that the maternal effect of PUM1 does not have a fully penetrant phenotype of absolute preimplantation lethality.

## Conclusion

In conclusion, our study shows that *Pum1* is a mammalian maternal effect gene similar to its *Drosophila* counterpart and that mammalian maternal PUM1 functions as a post-transcriptional regulator of mRNA during oocyte-to-embryo transition. We demonstrated here that one mechanism of action of PUM1 is by regulating mRNA stability. However, its role in translational regulation is not addressed by our study due to limited materials. Despite this, our study uncovers different developmental networks regulated by PUM1 and contributes to a broader understanding of how maternal proteins can shape the cytoplasmic contents of the oocyte and achieve cytoplasmic maturation.

## Additional files

**Additional file 1: Fig. S1.** A–D Shows the comparison of RNA-seq data with previous microarray study of mRNA transcripts changed from GV to MII (Su et al. [3]). *p < 0.02.

**Additional file 2: Fig. S2.** Two-cell m−z− and m−z+ embryos have more similar transcriptomes to each other than to m+z+ embryos. RNA seq analysis was performed on m+z+ (WT) two-cell embryos from *Pum1*^+*/*+^ reciprocal matings, m−z− (KO) two-cell embryos from *Pum1*^−*/*−^ reciprocal matings and m−z+ (HET) two-cell embryos from *Pum1*^−*/*−^ females mated with *Pum1*^+*/*+^ *male*. A The heatmap of Spearman correlation coefficient between the different oocytes and two-cell embryos. B–D Shows the scatterplot for the comparisons of the different two-cell transcriptomes.

**Additional file 3: Fig. S3.** Maternal and zygotic PUM1 are required for postnatal survival. The top panel shows the crosses observed over at least a 6 month period. n = number of matings pairs. %P0/P1 lethality is the number of pups that are born dead at birth or after 1 day after birth. Pups/litter–mean (SD).

**Additional file 4: Table S1.** List of the genes significantly differentially expressed between WT and Pum1-KO MII oocytes and had at least one PRE in their 3′UTRs.
