# Supplementary figures and images for: A role of Pumilio 1 in mammalian oocyte maturation and maternal phase of embryogenesis
Source: Cell Biosci. 2018 Oct 19;8:54. doi: 10.1186/s13578-018-0251-1 (PMC6194604; doi:10.1186/s13578-018-0251-1)

Fig. S1

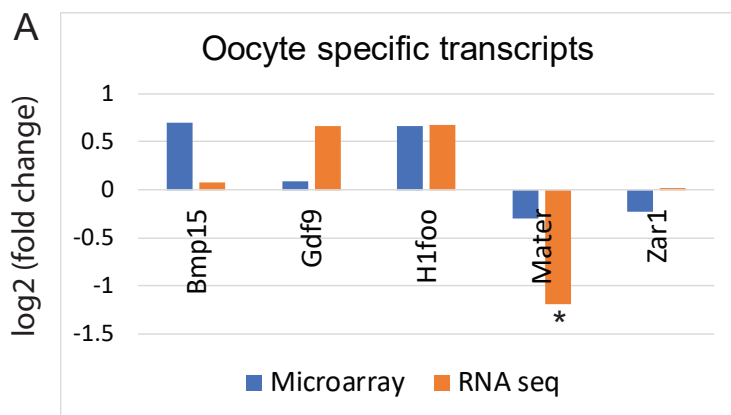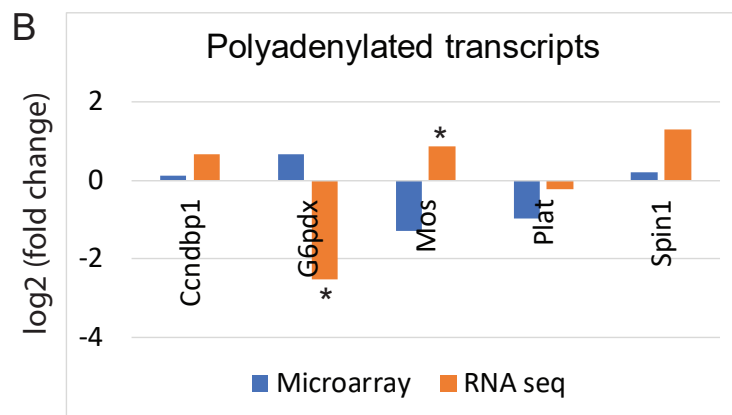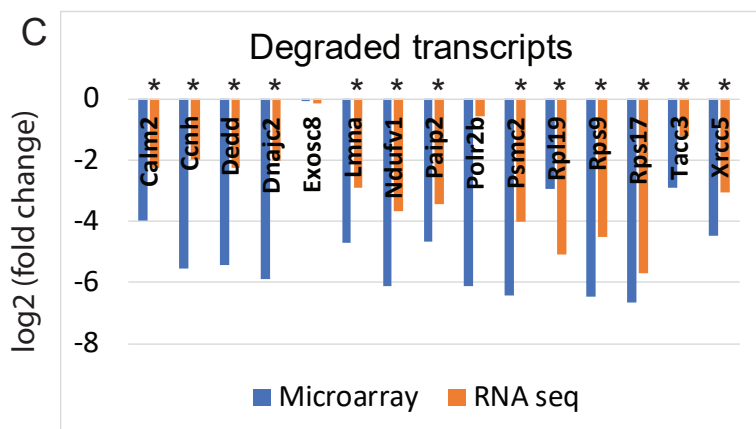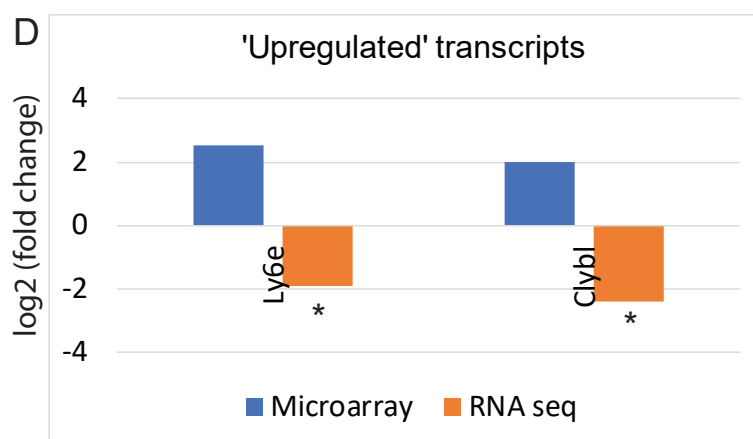

Supplement: Supplementary file 1 — Additional file 1: Fig. S1. A–D Shows the comparison of RNA-seq data with previous microarray study of mRNA transcripts changed from GV to MII (Su et al. [3]). *p < 0.02. [file 13578_2018_251_MOESM1_ESM.pdf]

Fig. S2

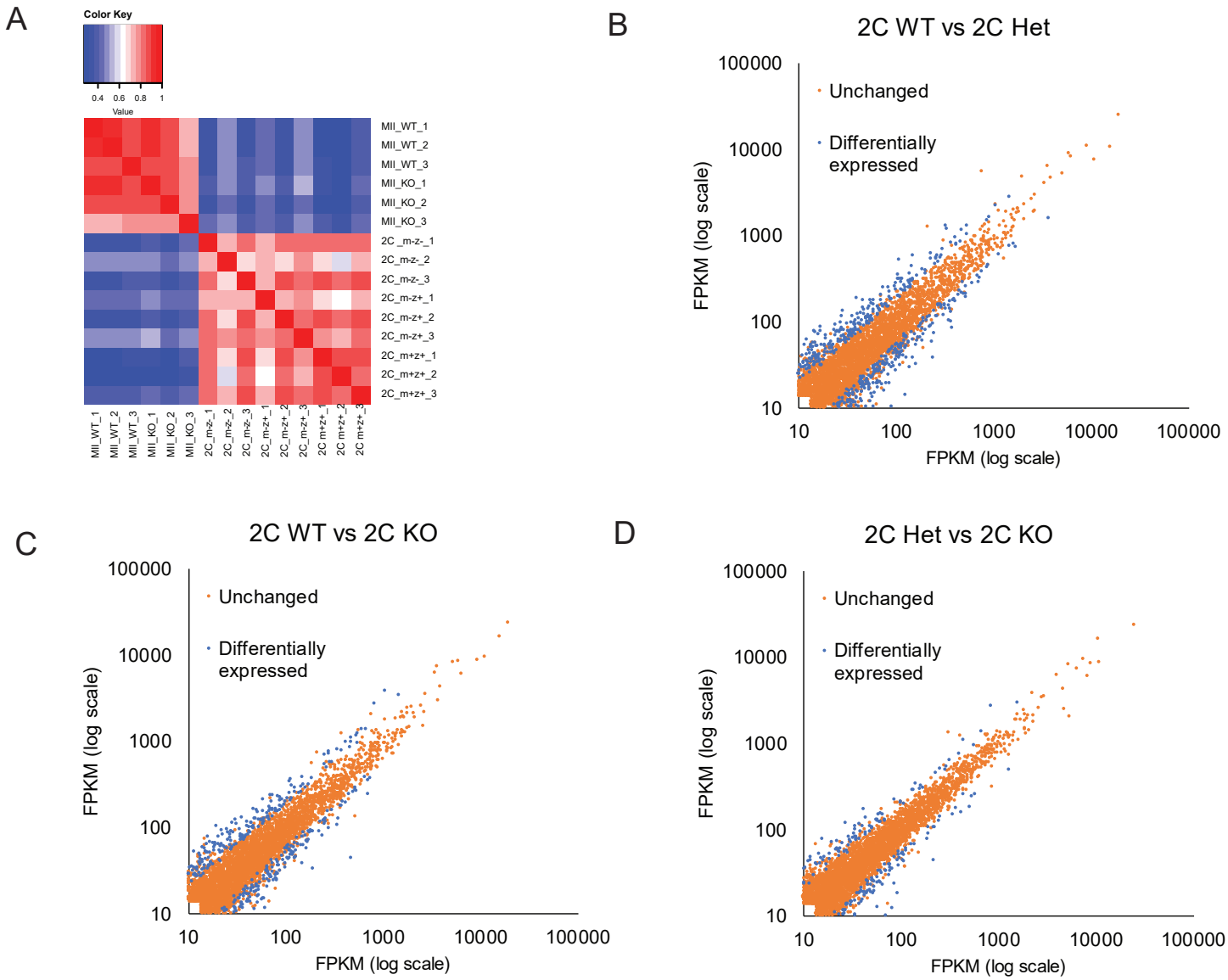

Supplement: Supplementary file 2 — Additional file 2: Fig. S2. Two-cell m−z− and m−z+ embryos have more similar transcriptomes to each other than to m+z+ embryos. RNA seq analysis was performed on m+z+ (WT) two-cell embryos from Pum1+/+ reciprocal matings, m−z− (KO) two-cell embryos from Pum1−/− reciprocal matings and m−z+ (HET) two-cell embryos from Pum1−/− females mated with Pum1+/+ male. A The heatmap of Spearman correlation coefficient between the different oocytes and two-cell embryos. B–D Shows the scatterplot for the comparisons of the different two-cell transcriptomes. [file 13578_2018_251_MOESM2_ESM.pdf]

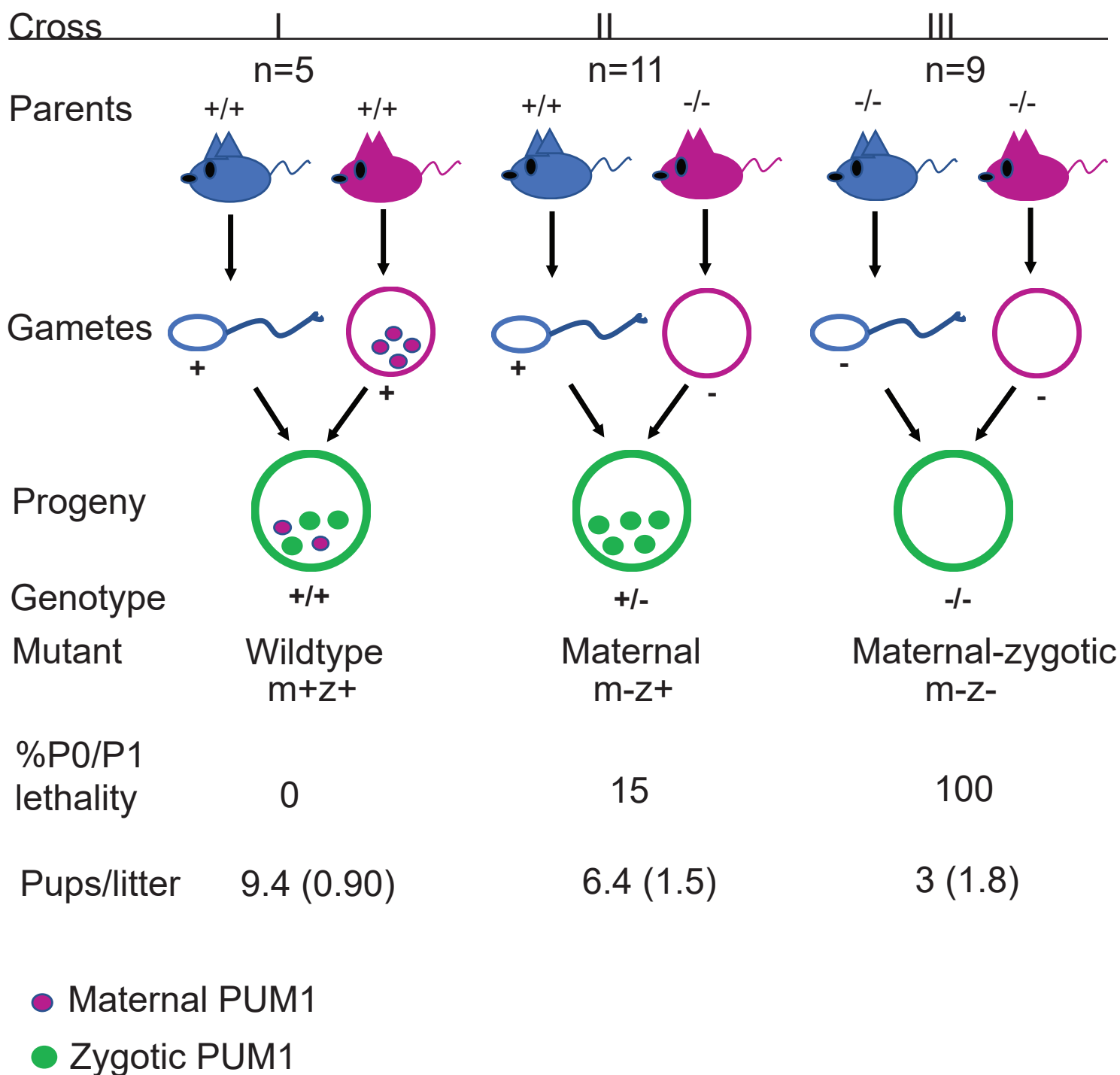

Fig. S3

Supplement: Supplementary file 3 — Additional file 3: Fig. S3. Maternal and zygotic PUM1 are required for postnatal survival. The top panel shows the crosses observed over at least a 6 month period. n = number of matings pairs. %P0/P1 lethality is the number of pups that are born dead at birth or after 1 day after birth. Pups/litter–mean (SD). [file 13578_2018_251_MOESM3_ESM.pdf]
