# Supplementary material for: A role of Pumilio 1 in mammalian oocyte maturation and maternal phase of embryogenesis
Source: Cell Biosci. 2018 Oct 19;8:54. doi: 10.1186/s13578-018-0251-1 (PMC6194604; doi:10.1186/s13578-018-0251-1)
Supplement: Supplementary file 4 — Additional file 4: Table S1. List of the genes significantly differentially expressed between WT and Pum1-KO MII oocytes and had at least one PRE in their 3′UTRs. [file 13578_2018_251_MOESM4_ESM.pdf]

Table S1 List of the genes significantly differentially expressed between WT and Pum1- KO MII oocytes and had at least one PRE in their 3'UTRs.

Lower levels in KO MII	Log2(fold change)	Higher levels in KO MII	Log2(Fold change)
Gm19757	-4.87927	*Cdk1*	4.64042
Zfp708	-3.53458	*Stk32a*	2.57811
Casc4	-1.5398	*Lancl1*	2.45338
Luc7l2	-1.37811	*Psmf1*	2.21055
Rassf5	-1.34017	*Rmdn3*	3.38138
Cflar	-1.40479	*Tmem60*	1.41618
Mtfr2	-1.12956	*Cd9*	2.33418
Kdm6b	-1.26677	*Rpl5*	1.55795
Abl2	-1.21962	*Rpl19*	2.35957
Fgfr1op	-2.1184	*Mfsd2a*	2.38774
Gm16702	-1.42897	*Cep41*	1.8403
Paxip1	-1.44875	*Dnajb1*	2.10664
Tyr	-1.70109	*Phf5a*	1.20871
Dcaf5	-1.5433	*Ppme1*	1.35459
Hiatl1	-1.15622	*Spc25*	1.24069
Ppfibp1	-1.36404	*Irf1*	1.49803
Mfsd11	-1.51214	*Rpl15*	1.48613
Cxcr2	-1.49832	*Arid5b*	1.23134
Slc25a32	-1.12659	*Kat8*	2.00157
		Kpna2	1.02854
		Eif2s1	1.21307

		Mea1	1.38465
		Usp42	1.17173
		Bcar3	1.41937
		Foxj3	1.05385
		Snupn	1.41138
		Lamtor5	1.49563
		Tmem29	1.70578
		Mfap1a	1.023
		Tox3	1.10131
		Ska3	1.78348
